# The genetic diversity and structure in the European polecat were not affected by the introduction of the American mink in Poland

**DOI:** 10.1371/journal.pone.0266161

**Published:** 2022-09-28

**Authors:** Begoña Martínez-Cruz, Hanna Zalewska, Andrzej Zalewski

**Affiliations:** Mammal Research Institute Polish Academy of Sciences, Białowieża, Poland; University of Warsaw, POLAND

## Abstract

The introduction and expansion of an invasive non-native species could have important consequences for the genetic patterns and processes of native species, moreover if the new arrival competes strongly for resources and space. This may result in the demographic decline of the native species. Knowing the effects on the levels of genetic diversity and structure in native species is key in terms of their conservation. We analysed temporal (over 50 years) genetic variation of the population of the European polecat (*Mustela putorius*), a species under threat in several European countries, in the Białowieża Primeval Forest (BPF), Poland, before and after the invasion of the American mink (*Neovison vison*). Using 11 microsatellite loci and a fragment of the mitochondrial control region we show that levels of diversity changed in the polecat population over 53 generations (over the period 1959–2012) and after the invasion of mink. When compared with other threatened European polecat populations, high levels of diversity are observed in the population in BPF in both periods, as well as in other areas in Poland. Our data shows that genetic structure was not present either before or after the mink invasion in BPF. This would suggest that the polecat population in Poland was not affected by invasive species and other negative factors and would be a potential good source of individuals for captive breeding or genetic rescue conservation management actions in areas where such actions are needed, for example the UK.

## Introduction

Invasive non-native species have the potential to cause a decrease in native species number and density in ecosystems mainly by predation or competition [1]. If the introduction of the alien causes a demographic decline in any native species it may result in deleterious consequences for its genetic diversity and structure, which would be difficult to assess. Only the study of the population pre-dating the arrival of the alien provides the baseline to which directly compare the pattern extant in the population at present and infer the actual effect of the perturbation whenever other confounding factors could be discarded [2]. This kind of temporal approach has shed light on the processes and patterns affecting genetic diversity after the occurrence of external perturbations to a population, at both historical (e.g. [3, 4]) and evolutionary scales (e.g. [5, 6]) that would otherwise be difficult to infer.

Native to North America the American mink, *Neovison vison*, is a small generalist semi-aquatic mustelid that was brought to Europe in the 1920s for commercial fur farming [7]. Escapees from farms colonized wild habitats in many West European countries [7]. Since the 1930s, thousands of American mink were also deliberately released into the wild in the former Soviet Union to form a harvestable population [8]. As a consequence of these two pathways of introduction, the European range of the American mink extended very quickly [9] and at present it inhabits many riparian and mesic habitats in the vast majority of European countries [7, 10, 11]. Although the catastrophic effect of the American mink invasion on the European mink is well documented [12–14], less attention has been paid to its negative effects on other species like the European polecat, *Mustela putorius*, despite being the carnivore species most affected by it [15].

The European polecat is a medium-sized mustelid widespread in the western Palaearctic [16]. It inhabits a wide range of habitats, including riparian habitat, bog and deciduous forests and grasslands, as well as rural areas including farms and villages [17–19]. In these wild range of habitats polecats feed on a wide spectrum of prey including small mammals, rabbits, birds, reptiles, amphibians and ungulate carcasses [17, 20]. Although classified as a least concern species [21] its populations have been declining over the last few decades in most European countries due to habitat alteration as the drainage of wetlands and changes in the agricultural landscape, direct persecution, prey decline, hybridization with ferrets and the invasion of the American mink [22–26].

The American mink and polecats’ spatial and trophic niches partially overlap [27, 28] potentially resulting in strong competition between both species especially during periods of low food abundance (winter or drought). The spread of the American mink was accompanied by a significant reduction in polecat density in some regions [15, 24, 26] and by the occurrence of a male-biased sex ratio in polecat populations [29–32]. Whether these alterations may have affected the genetic patterns of the polecat populations in terms of diversity and structure, is still to be disentangled. The Białowieża Primeval Forest (BPF) in NE Poland represents an excellent scenario where to investigate such impact.

BPF is one of the last non-altered habitats in Europe, with low hunting or poaching pressure on small mustelids, a high density of polecat preys and with no ferrets or European mink living there (thus free of potential hybridization polecat—related species) [33]. The American mink population was established in BPF in the late 1970s [9, 10, 34] and since then the population has been increasing in size and its density has reached eight individuals per 10 km of river bank on some rivers [28]. After the American mink invasion, the polecat was pushed mainly to small streams and bog ash-alder forest, as the American mink prefers medium-size rivers [28]. However, the increasing American mink populations also colonized some of the small streams from where the polecat density decreased or the species disappeared [30]. Forty years after the American mink invasion however, both species still coexist in BPF, despite the very high food niche overlap between both predators [35].

In this study we used eleven autosomal microsatellite markers and a fragment of the mitochondrial DNA to investigate the effects of the American mink invasion on the neutral genetic diversity of the polecat population in the Białowieża Primeval Forest. We genotyped the historical (pre-American-mink-arrival) and current (post-American-mink-arrival) polecat populations in BPF to analyse: i) potential changes in the levels and distribution of genetic diversity due to the American mink invasion, and ii) changes in the demographic history of the polecat population. Additionally, we genotyped samples from other locations in order to understand the current genetic structure of the polecat population in Poland.

## Material and methods

### Sample collection, DNA extraction and amplification

**Fig 1:**
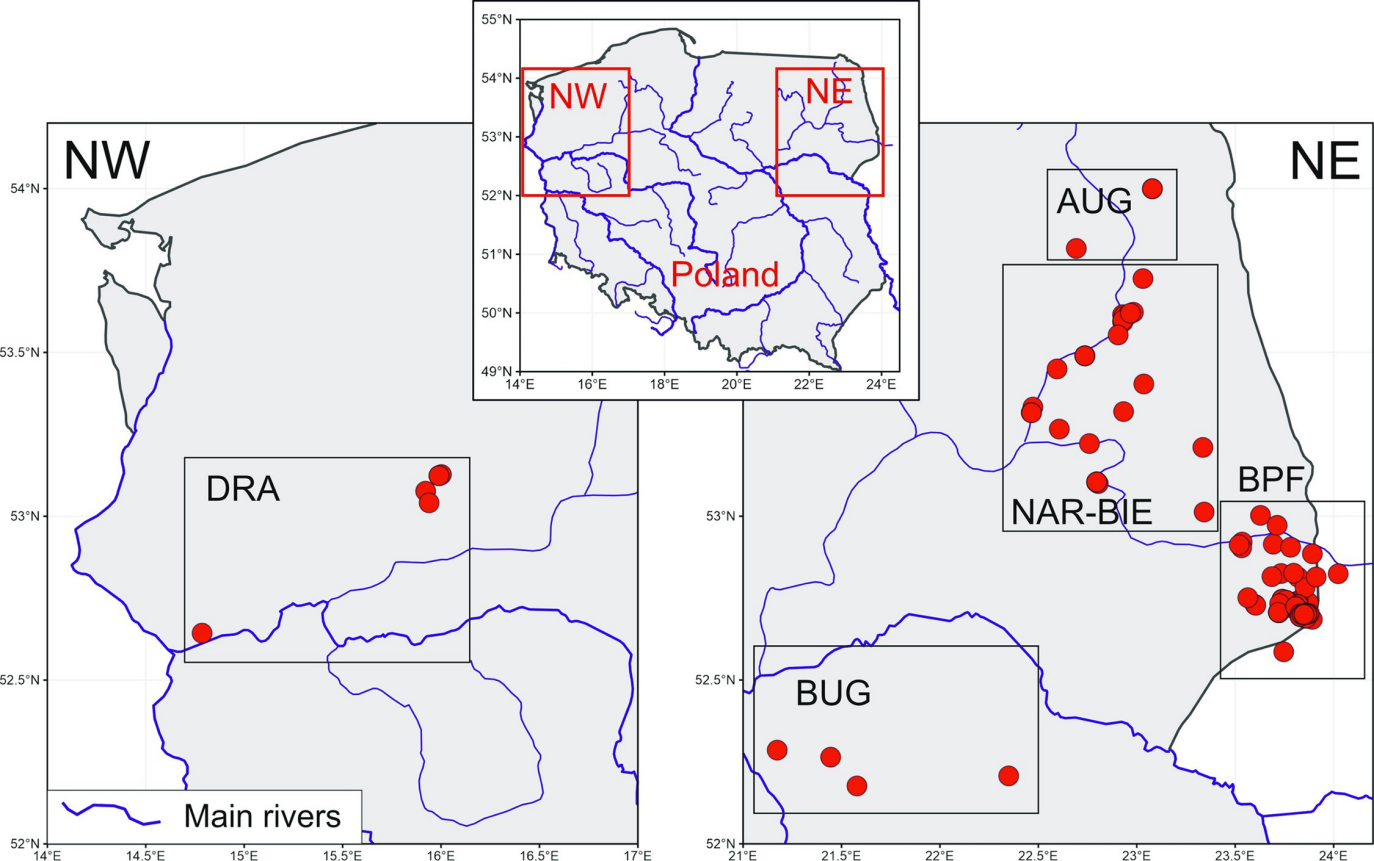
Map with the sample localities. AUG–Augustów Forest; NAR-BIE–Narew and Biebrza river basins; BPF–Białowieża Primeval Forest; BUG–Bug river basin; DRA–Drawa and Warta river basins. Background map: Natural Earth (public domain).

A total of 92 individuals were collected during the period between 2000 and 2012 from the areas of Białowieża Primeval Forest (BPF, n = 51, including two samples from Belarussian part of BPF), Biebrza river basin (BIE, n = 20), Narew river basin (NAR, n = 8), Augustów Forest (AUG, n = 3), Drawa and Warta river basins (DRA, n = 5) and Bug river basin (BUG, n = 4) (contemporary samples) and one with unknown location (OTH) (Fig 1). Muscle or ear samples were collected from occasional road casualties or animals found dead in the forest or in the villages (all sites). The hair samples were collected from live-trapped polecats (in BPF) and polecats captured unintentionally during various projects studying American mink biology conduced in all sites (except AUG and BUG). This study did not require permits to approve the field site access. All samples were collected during other projects under permission granted by local and government authorities (Ministry of Environment Protection: DLOPiKog. 4201/143/00 and DLgl-6713-35/09/1399, Poviat Starosty in Hajnówka: RS6121/1/2000).

All tissue and hair samples were stored at -20°C prior to DNA extraction. Additionally, DNA samples were obtained from 116 museum skulls from BPF stored at the zoological collection of Mammal Research Institute PAS collected over the period 1959–1998, and originated from either road kills or animals shot by hunters. Material from historical samples was obtained from the turbinals in the nasal cave. In the case of BPF, both contemporary and museum samples combined represents a time span of 50 years. (See S1 Table for a complete description of the samples). Following the approach described by [36] and under the hypothesis of a strong effect of the harsh weather in the polecat population dynamics, we checked the temporal stability of the genetic frequencies in BPF. With the mean lifespan of the polecat of between 3 to 4 years (maximal life span is 8 years), and the age of maturation one year [37], and being a species with overlapping generations, we could define a cohort in periods of four years. Consequently, samples from BPF were subdivided in different cohorts comprising all individuals sampled within periods of four years. We used a one-year sliding window strategy in order to account for any unexpected variance. Only samples with n ≥ 8 individuals were considered for the analyses. Additionally, we could define a cohort integrated by individuals from NAR-BIE sampled between 2008–2011 (n = 21 in the case of mtDNA; n = 25 in the case of microsatellites) in order to test for spatial fluctuations (S2 Table).

DNA from fresh tissues (mussel, ear, and hair) was extracted using the DNA Tissue Kit (QIAGEN) following the manufacturer’s instructions. Samples of the turbinals were extracted from the nasal cave with the help of a stick. Due to the softness of this bone tissue, each sample could be powdered manually inside the tube used for its digestion by using a plastic stick. DNA was extracted using the QIAamp DNA Investigator Kit (QIAGEN) following the manufacturer’s instructions. Bone extractions were carried out in a separate room specially conditioned for working with non-invasive and ancient material. All the manipulations were made in a laminar-flow hood that was UV irradiated previous to and after any manipulation. Negative extraction controls were performed in every extraction of 23 samples to monitor possible contamination. Among the 150 museum samples, 92 rendered enough DNA for the microsatellite study (77%) and 63 were sequenced for the mtDNA.

Samples were genotyped with twelve microsatellite markers. Six of them were developed for the American mink (Mvi57, Mvi111, Mvi1006, Mvi1341, Mvis002 and Mvis072), five of them were developed for the polecat (MP07, MP22, MP28, Mp3.1 and Mp3.18), and one was developed for the European mink, *M*. *lutreola* (MLUT25). Twelve additional microsatellite markers developed for the American mink had also been assayed in an initial set of 18 random samples and discarded due to spurious results (see S3 Table for a complete description of the markers and references).

With the set of twelve microsatellite that rendered good results, three different multiplex were carried out using a Multiplex PCR Kit (QIAGEN) in final reaction volumes of 5 μl for the fresh samples and 8 μl for samples extracted from turbinals, containing a final concentration 1x QIAGEN Multiplex PCR Kit (Qiagen), (0.2 μM) of each primer, 0.25 μg/ μl BSA (Fermentas) and 30 ng of DNA (for museum samples 2 μl of DNA extraction were used) in a (DNA Engine Dyad^®^ BIO-RAD) thermocycler. PCRs consisted of an initial denaturation step at 95°C for 15 minutes, followed by 30 cycles (33 cycles for museum samples) of 94°C for 30 s, 60°C for 90 s, and 72°C for 60 s, and a final extension at 60°C for 30 min. To monitor the possibility of allelic dropout related to low DNA quality and/or quantity in museum and hair samples these were amplified and genotyped three times each and for each multiplex. A high rate of allelic dropout was observed for the Mvis072 marker in samples from the museum so this marker was excluded from all analyses. A final dataset of eleven microsatellite markers was used for further analysis. Fragments were analysed in an ABI3130xl Genetic Anlyser (Applied Biosystems). Allele scoring was performed with the GeneMarker (v 1.85) software (SoftGenetics). Microsatellite genotypes are provided in S4 Table.

A fragment of 640 bp of the mtDNA control region was amplified in forward and reverse orientations using the primers LutF [25] and Mpu1R (5’-tgtgtgatcatgggctgatt-3’–the later designed with the help of Primer3 software. PCRs were performed in a final volume of of 10 μl (fresh samples) or 20 μl (museum samples) and comprised 3 μl (fresh samples) and 6 μl (museum samples) of DNA extract (3–10 ng—about 60 ng per reaction), 5 μl (fresh samples) and 10 μl (museum samples) HotStar Taq Master Mix Kit (Qiagen), 0.5 μM of each primer in final concentration. PCR cycling comprised an initial denaturation at 95°C for 15 min, followed by 35 (fresh samples) and 38 (museum samples) cycles at 94°C for 60 s, 56°C for 60 s, 72°C for 90 s and a final extension at 72°C for 10 min. PCR products were sequenced using the BigDye v 3.1 Terminator Cycle Sequencing Kit (Applied Biosystems) and an automated DNA fragment analyser (ABI-3130xl; Applied Biosystems, Inc.). Sequences were edited, assembled and aligned using the program Sequencher 4.9 (Gene Codes Corporation).

### Data analyses

Only the cohorts defined in BPF and NAR-BIE were considered to have adequate sizes to perform population analyses on them. Samples from AUG, DRA and BUG (n ≤ 5 in all cases) were considered only in the analysis of population structure.

#### Mitochondrial DNA analyses

*Diversity*. Haplotype and nucleotide diversity (π) and the number of segregating sites (S) were estimated and mtDNA haplotypes identified for the global polecat populations with DnaSp v5 [38]. Indices of genetic diversity were computed in ARLEQUIN 3.5 [39].

*Population differentiation and genetic structure*. We calculated ΦST and exact test of differences between the population pre and the population post arrival of the American mink (excluding the individuals from 1974 to 1997, as indicated before) using Arlequin 3.5 [39]. In order to test for fluctuations in the genetic frequencies through time we calculated pairwise ΦST and pairwise differences among all cohorts defined in BPF for every sliding window dataset. The potential effect of the arrival of the American mink on the gene frequencies of the polecat population was tested through an analysis of molecular variance (AMOVA) by pooling cohorts from BPF into pre- or post- American mink arrival for every sliding-window dataset using Arlequin 3.5. Any individual sampled between the years 1974 and 1997 was excluded from the AMOVA analyses as they belonged to the cohorts present during or immediately after the arrival of the American mink. We also included the cohort defined in NAR-BIE to test for contemporary fluctuations in space. All p values were corrected after Bonferroni to account for multiple comparisons. The significances of the observed ΦSTs were tested using 5000 random permutations of the data matrix.

*Population demographic history*. Past population dynamics of the polecat was investigated with a Bayesian Skyline Plot model using BEAST v1.8.1 [40]. We used a mutation rate of 7.244 x 10^−8^ substitutions/site/generation as estimated for *Mustela erminea* in [41] from a combined set of mitochondrial regions. To construct the BSP we assumed an HKY+G model of evolution, and a strict molecular clock which is the more robust method when analysing intraspecific data. Three MCMC chains from different starting points were run, each based on 30,000,000 generations, sampled every 1,000, with the first 3,000,000 discarded as burn-in. Visual inspection of the MCMC autocorrelation plots with Tracer v1.7 [42] showed that runs had converged to the equilibrium distribution. All runs had an effective sample size (ESS) of minimum 500.

#### Microsatellite markers

*Diversity*. Observed and expected heterozygosities (H_o_ and H_e_ respectively) and heterozygote deficits, linkage disequilibrium and Hardy-Weinberg (HW) equilibrium were tested for BPF and NAR-BIE with the probability test of Genepop v3.5 [43] using a Markov chain method to estimate without bias the exact p values of these tests [44]. Significance levels were adjusted with sequential Bonferroni correction in order to correct for the effect of multiple tests [45]. Genetic diversity within groups was estimated as the number of allele per locus (k), and the allelic richness (AR) using FSTAT [46]. Levels of allelic richness and He between groups were compared using a Wilcoxon sign-rank test.

*Population differentiation and genetic structure*. We calculated F_ST_ and exact test of differences between the population pre and the population post arrival of the American mink using Arlequin 3.5. Using the same scheme of sliding windows as for mtDNA, we calculated pairwise F_ST_ value [47] among all the cohorts defined in BPF for every sliding window dataset using Arlequin 3.5. We also included the cohort defined in NAR-BIE to test for contemporary fluctuations in space. All p values were corrected after Bonferroni to account for multiple comparisons. Following a similar scheme than for mtDNA, AMOVA were performed using Arlequin 3.5. We preferred F_ST_ to R_ST_ distances as the latter would underestimate differentiation when populations are not highly structured [48] whereas frequency-based estimates have been shown to be more appropriate when comparing closely related populations [49]. Correlation analysis between genetic and geographical distances (Mantel test) between all pairs of contemporary individuals was calculated with GenePop 3.4 [50].

We further investigated the pattern of spatial genetic structure at present using data from microsatellites. For this we conducted a spatial principal component analysis (sPCA) for BPF (including individuals sampled in the period 2009–2012) and NAR-BIE site (including individuals sampled in the period 2008–2011) using the Adegenet (version) package [51] for R. The sPCA identifies cryptic spatial patterns of genetic structuring across the landscape, and accounts for spatial autocorrelation issues associated with neighbor-mating and sample distribution [52]. It uses Moran’s I index of spatial autocorrelation to compare allele frequencies observed in individuals at given spatial locations with those of individuals at neighbouring sites [51]. Two tests were used to detect global (e.g. clines and patches) and local structure (when spatially close individuals are genetically dissimilar).

We performed a clustering analysis of all the individuals sampled in this study using STRUCTURE v2.3 [53]. This version allows structure to be detected at lower levels of divergence than the original model. Values of K = 1 to K = 7 were tested. In a second step, we also performed the clustering analyses including only all individuals from BPF, testing values from K = 1 to K = 5. To ensure consistency of the results, we performed 10 independent runs per K tested in all cases. Each Markov chain was run for 10^6^ steps after a burn-in period of 10^5^ steps. All chains were run using the *F* model for correlations of allele frequencies across clusters [54]. Any possible genuine multi-modality of the data was investigated using the Greedy algorithm implemented in CLUMPP [55]. The figure was drawn using the package CLUMPAK [56].

*Population demographic history*. The occurrence of a genetic bottleneck in the recent past was investigated in the contemporary sample using the program BOTTLENECK [57]. The program was run under the two-phase model that is supposed to fit microsatellite evolution better [58] i.e. with 95% and 78% of the stepwise mutation model with a variance for mutation size set to 12, following what has recently been performed for the same species [22]. Additionally, the method assesses the distribution of allele frequencies, expected to be L-shaped under mutation-drift equilibrium [59]. We estimated the effective population sizes of the populations pre- and post-mink arrival in BPF using the linkage disequilibrium (LD) method [60], as implemented in NeEstimator V2.1 [61]. The demographic history was further investigated using the hierarchical Bayesian model based on Markov Chain Monte Carlo (MCMC) and coalescent theory implemented in MSVAR v1.3 [62, 63]. This method has been proven to precisely estimate the parameters that characterize the demographic history provided that the change in population size was neither too recent nor too weak, and to outperform both the M-ratio test [64] and the BOTTLENECK method [59] in the detection of population declines [65]. It allows the estimation of current and ancestral population sizes (N_1_ and N_0_, respectively), the mutation rate (μ) and the time since the population started changing (T). We used wide lognormal priors with large variances to affect posterior distributions as little as possible (as used in [66]). A generation time of 1 year was assumed [25]. Three independent runs from different starting points and combinations of priors and hyper-priors were conducted in order to check the robustness of the results by assessing the convergence of the chains. We ran a total of 5 x 10^9^ iterations thinned by 2 x 10^5^, and the first 10% was eliminated as burn-in. A final amount of 5 x 10^4^ sampling points was used to build the posterior distributions. We assessed convergence with the Gelman-Rubin statistic, we calculated the 95% High Posterior Density (HPD) intervals and plotted the results using the R packages Coda [67] and Locfit [68]. Once convergence was assessed, we combined the three chains to get a final dataset of 15 x 10^4^ sampling points to build a single, definite posterior distribution. We visualized the trace plots of the MCMC chains and calculated the mean values and the 90% high probability density (HPD) of the posterior distribution for each parameter with the program Tracer 1.7 [42].

## Results

### Diversity

#### Mitochondrial DNA

We obtained sequences from the control region in 149 samples from all locations. Among them, 45 were from before (all of them from BPF) and 84 were from after the American mink invasion (48 of them from BPF), 17 corresponded to individuals sampled in between (period 1974–1997), and three were from unknown date and/or origin. All sequences were deposited in GenBank under accession numbers OK644317-OK644331 and OM069433—OM069566. The length of the fragment was 532bp excluding the previously described polypyrimiden CnTn tract in the control region of polecats [22, 69, 70]. Those identified 17 segregating positions and 15 haplotypes, Hp1 to Hp15 (S5A Table). Six of them were found in a single individual (one of them in post-American mink BPF, one in pre-American mink BPF, one in the period in between in BPF, two in NAR, and one in BUG). Eight haplotypes were found in the population before the arrival of the American mink (eleven occurred in post-American mink BPF) whereas twelve where found in the post-American mink population (nine if we take only into account the individuals from BPF). The overall mean haplotype and nucleotide diversities and the average number of nucleotide differences were 0.75, 0.0034 and 1.83, respectively. The most common haplotype, Hp1, was shared by 47% of the individuals (60 out of 129). Apart from the singletons, only haplotypes Hp3 (n = 5), Hp7 (n = 3) and Hp13 (n = 10) were private from a single location, BPF (with H13 happening in pre-American mink BPF, one in the BPF in the period in between, and two in the post-American mink BPF population). Haplotype and nucleotide diversity (H_D_ and ∏) in the polecat population were 0.82 and 0.0043 before, and 0.67 and 0.0028 after the arrival of the American mink (Table 1). This points to a reduction in the levels of mtDNA genetic diversity after the arrival of the American mink. This can be due to a more skewed distribution of haplotypes in the current population (see S5B Table). This difference still appears when considering only the individuals from BPF in the population after the arrival of the American mink (H_D_ = 0.72, ∏ = 0.00278, Table 1).

**Table 1:** Mitochondrial diversity in the European polecat before and after American mink invasion. *n* stands for the number of samples; *N pol*. *sites*, number of polymorphic sites.

Population	n	N haplotypes	N pol. sites	Haplotype diversity	Nucleotide diversity	Mean number of pairwise differences
Before American mink	45	8	9	0.822±0.029	0.0043±0.0027	2.273±1.272
All after American mink	84	12	13	0.672±0.052	0.0028±0.0019	1.502±0.914
BPF after American mink	49	11	10	0.725±0.058	0.0029±0.002	1.546±0.941

#### Microsatellites

The genotyping of the whole set of individuals (n = 208) with eleven markers rendered a total of 84 alleles. Number of alleles per locus ranged between five and ten, and global expected heterozygosity H_e_ was 0.65. The genotyping of the pre-American mink population (n = 88 individuals, all of them from BPF) with eleven markers rendered a total of 74 alleles. Number of alleles per locus ranged between four and ten and expected heterozygosity H_e_ was 0.63. The genotyping of the post-American mink population (n = 92 individuals) with eleven markers rendered a total of 80 alleles. Number of alleles per locus ranged between five and nine and expected heterozygosity H_e_ was 0.67. The genotyping of the BPF population after the arrival of the American mink (n = 51 individuals) with eleven markers rendered a total of 73 alleles. Number of alleles per locus ranged between four and nine and expected heterozygosity H_e_ was 0.66. Deviation from Hardy-Weinberg equilibrium was not detected in the populations (p > 0.05). When compared to the total post-American mink population, He was lower in historical BPF (Wilcoxon signed rank test, p < 0.001), and AR was similar (Wilcoxon signed rank test, p = 0.083). Expected heterozygosity was similar between the pre- and post-arrival mink population in BPF (Wilcoxon signed rank test, p = 0.067) and AR was lower in the historical population (Wilcoxon signed rank test, p = 0.032) (see Table 2). Finally, both H_e_ and AR were similar between contemporary BPF and BIE-NAR populations (H_e_ 0.63 and H_e_ = 0.66 Wilcoxon signed rank test, p = 1.00; and p = 0.083 for AR comparisons).

**Table 2:** Microsatellite genetic diversity in the European polecat population.

	Before American mink invasion (n = 88)					All after American mink invasion (n = 92)				BPF after American mink invasion (n = 51)			
Locus ID	N_A_	A_R_*	A_R_**	H_o_	H_e_	N_A_	A_R_*	H_o_	H_e_	N_A_	A_R_**	H_o_	H_e_
Mvi100	8	8.000	7.846	0.766	0.793	9	8.748	0.807	0.799	8	8.000	0.792	0.798
Mvi111	5	4.999	4.879	0.674	0.656	6	5.977	0.593	0.692	6	5.959	0.600	0.609
MP07	6	5.998	5.849	0.500	0.490	6	5.996	0.478	0.515	6	5.941	0.529	0.518
Mvi002	6	5.998	5.866	0.534	0.533	8	7.511	0.489	0.566	5	5.000	0.451	0.523
Mvi57	5	5.000	4.959	0.568	0.529	5	5.000	0.576	0.637	5	5.000	0.529	0.603
MP28	9	8.985	8.714	0.705	0.685	9	8.948	0.772	0.792	9	8.879	0.804	0.776
MLUT25	4	3.985	3.795	0.455	0.434	5	4.674	0.489	0.479	4	3.941	0.549	0.535
Mp3.1	10	9.735	8.876	0.795	0.793	9	8.837	0.804	0.845	9	8.941	0.863	0.871
MP22	7	7.000	6.977	0.795	0.773	8	7.829	0.793	0.790	7	6.997	0.784	0.777
Mvi134	8	7.735	6.827	0.625	0.599	8	7.936	0.543	0.613	7	6.935	0.490	0.598
Mp3.18	6	5.882	5.375	0.655	0.613	7	6.966	0.696	0.634	7	6.935	0.706	0.637
All	74	6.665	6.360	0.643	0.627	80	7.129	0.640	0.669	73	6.593	0.645	0.659

*n* number of samples; *N*_*A*_ number of alleles; *A*_*R*_ allelic richness; *H*_*o*_ observed heterozygosity; *H*_*e*_ expected heterozygosity.

*Based on 77 individuals (comparison with the All after American mink invasion).

**Based on 48 individuals (comparison with the BPF after American mink invasion).

#### Population differentiation and genetic structure

*Mitochondrial DNA*. The ΦST value between the pre and post arrival of the American mink populations was low but significant (ΦST = 0.08 p < 0.001). P value for the exact pairwise differences was as well significant (p < 0.001). This pattern was the same when considering only the individuals from BPF in the population post (ΦST = 0.07 p value < 0.001) and a significant p value for the exact pairwise differences (p < 0.001). This difference might be explained by the change in haplotype frequencies. Only nineteen out of 74 pairwise comparisons among cohorts in BPF and NAR-BIE sites were significant after Bonferroni correction for both ΦSTs and pairwise differences (S6 Table). Significant differences seem to particularly affect the cohorts by the end of the 60’s and the beginning of the 70’s. This is an interesting result as polecats and American mink started to coexist in the end of 70’s [9, 34]. AMOVA results indicated that the cohort explains a small although significant amount of the variance, but the group does not (the group being the populations pre- and post-American mink invasion). These analyses showed that the arrival of the American mink did not leave any imprint strong enough to be detected at the mitochondrial level, but for the shift in haplotype frequencies (S5B Table).

*Microsatellites*. The F_ST_ value between the pre and post arrival of the American mink populations was very low but significant (F_ST_ = 0.015 p < 0.001). Otherwise, the p value for the exact pairwise differences was not significant (p = 1.00). This pattern was the same when considering only the individuals from BPF in the population post arrival of the American mink (F_ST_ = 0.014 p < 0.001), and a very strong non-significant p value for the exact pairwise differences (p = 1.00). The exact test has been shown to be more powerful than the FST test [71], and this together with the very low F_ST_ value, suggest that if any differentiation is present it might be subtle. Thirty-five out of 98 pairwise comparisons among cohorts in BPF and NAR-BIE sites were significant after Bonferroni correction (S7 Table). As in the case of the mtDNA, there are some significant differences that mostly imply the cohorts by the end of the 60’s and the beginning of the 70’s, although no clear pattern of differentiation between the polecat populations pre- and post-American mink invasion emerges either with these markers. These slight differences could account for the significant F_ST_ p values when comparing the pre and post populations. As with mtDNA, the AMOVA results showed that the cohort explains a small although significant amount of the variance, but the group does as well in two out of the four waves defined by the sliding window. These results, suggest that the American mink could have had a temporal effect on the levels of genetic diversity but they did not leave an imprint strong enough to be detected by all the statistics applied here (Table 3). In addition, the polecat population in Poland does not show an IBD pattern (Mantel test, R = 0.07, p>0.99). As an alternative explanation, the markers used in this study might not be informative enough to unambiguously show the pattern of alterations that the invasion of the American mink produced in the genetic diversity of the polecat population.

**Table 3:** Analyses of AMOVA. Groups indicate pre- or post-American mink invasion populations. Populations in the table refer to the different cohorts.

		11 STRs	mtDNA
		(% variation)	(% variation)
Cohorts pre- (1959–1962), (1963–1966), (1967–1970), and post- (2000–2003), (2008–2011) American mink	Among groups	0.68, p = 0.12	0.05, p = 0.4
	Among populations within groups	1.06**	10.31**
	Within populations	98.28***	89.64***
Cohorts pre- (1960–1963), (1964–1967), (1968–1971), and post- (1997–2000), (2001–2004), (2009–2012) American mink	Among groups	0.96**	5.86, p = 0.12
	Among populations within groups	1.17***	2.73, p = 0.16
	Within populations	97.87***	91.40*
Cohorts pre- (1961–1964), (1965–1968), (1969–1972), and post- (1998–2001), (2002–2005) American mink	Among groups	1.07*	0.73, p = 0.1
	Among populations within groups	1.50***	13.62***
	Within populations	97.43***	85.66***
Cohorts pre- (1958–1961), (1962–1965), (1966–1969), and post- (1999–2002) American mink	Among groups	0.93, p = 0.15	2.44, p = 0.19
	Among populations within groups	1.78***	7.04*
	Within populations	97.30***	90.51***

P values as follows: * p < 0.05,

** p < 0.01,

*** p < 0.001.

**Fig 2:**
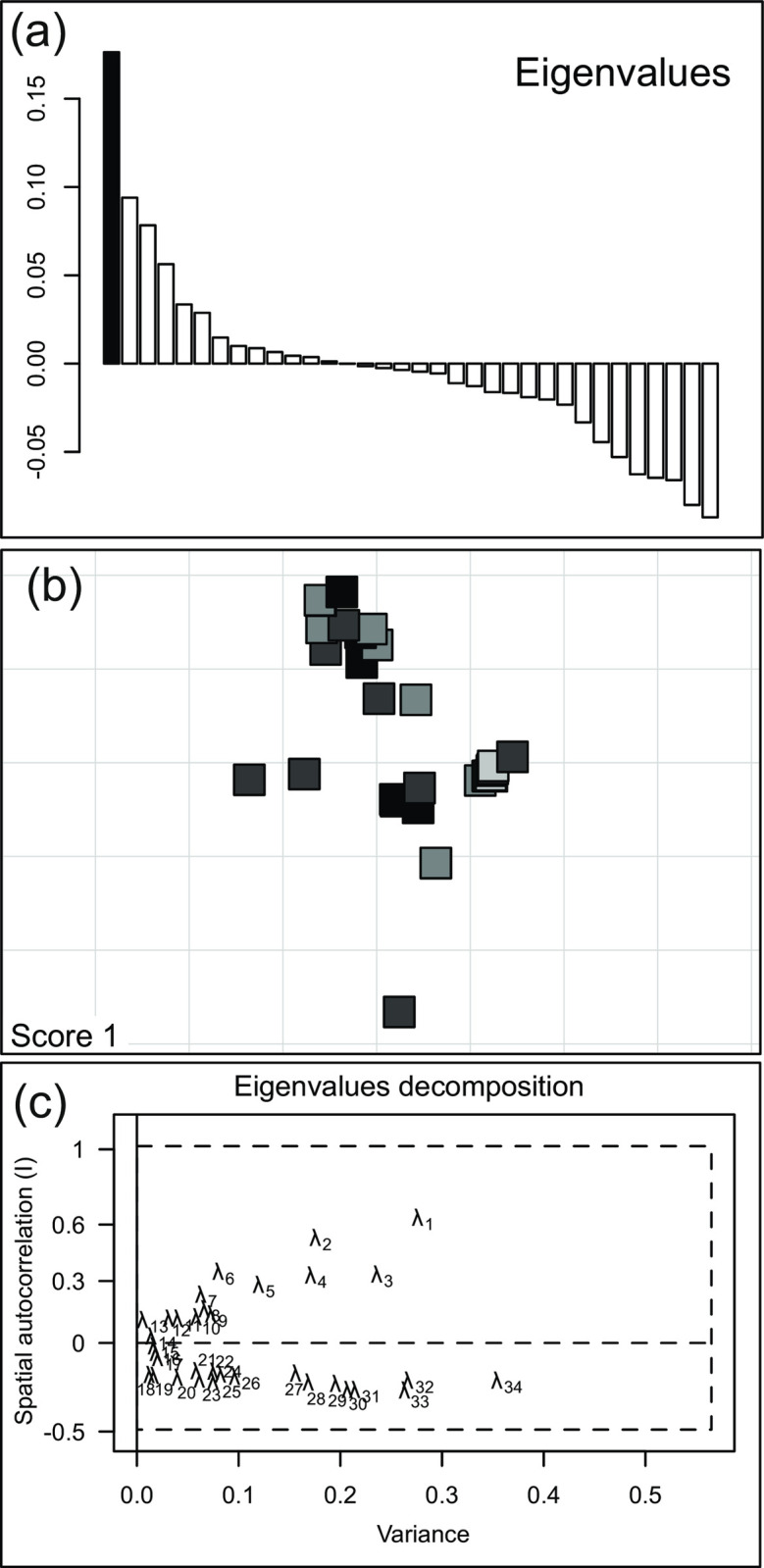
Results of the spatial principal component analysis (sPCA) for contemporary Białowieża Primeval Forest and Narew-Biebrza river populations based on 11 microsatellite loci. a) Eigenvalue plot and b) screenplot show that the first axe is the most important explaining the genetic variation that is not statistically significant (p < 0.05). c) Scores in space; grey levels are used for different absolute values with black and white being well differentiated.

**Fig 3:**
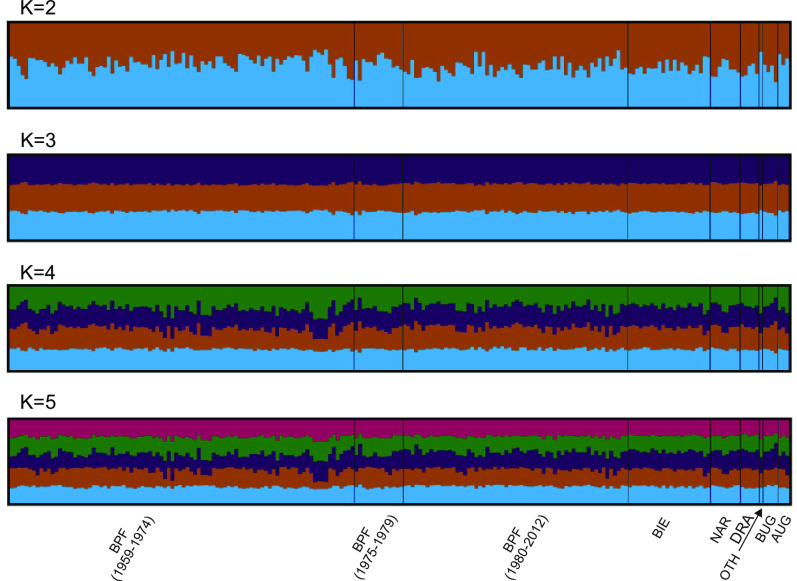
Population structure inferred from microsatellite data using the software package STRUCTURE. Each output represents the matrix of membership coefficients averaged over 40 independent runs with CLUMPP. Acronyms as in Fig 1.

The sPCA analysis including the contemporary BPF and NAR-BIE sites (Fig 2) showed no pattern of spatial structure at global or local scale (p = 0.27 and p = 0.74, respectively), in agreement with the non-significant F_ST_ value between them. The clustering analysis of all the individuals in BPF using the STRUCTURE algorithm did not show any pattern of structure. A similar absence of structure arose when the complete dataset was included in the analysis, comprising also individuals from AUG, DRA, BUG, and one individual from an unknown location (Fig 3).

#### Population demographic history

*Mitochondrial DNA*. The analyses with BEAST was not able to detect a bottleneck signal, but suggested that the N_e_ in the polecat population has been high and mostly stable for the last 17.5 Ky (S1 Fig).

*Microsatellites*. The analyses with BOTTLENECK showed that the observed proportion of heterozygotes was not significantly different to that expected under equilibrium for the observed number of alleles under the two-phase model (TPM) with a 95% of SMM model (P = 0.97, Wilcoxon test) or with a 78% of stepwise mutation model (SMM) (P = 0.82, Wilcoxon test). The mode shift test showed a normal L-shaped distribution in both cases. The analyses rendered almost identical results for the historical population in BPF. Estimated effective population size for the population pre-dating the arrival of the American mink in BPF (N_e_ = 74.1, CI 57.8–98.7) was similar than that of the population after (N_e_ = 75.9, CI 53.4–119.9).

Using the coalescent-based approach implemented in MSVAR 1.3 we found evidence of a historical decline in the polecat population size. An ancestral population with Ne of 15,382 individuals (191–32,359,365 HPD 95%) suffered a reduction that brought the population to a N_e_ of 2,399 individuals (11.75–223,872 HPD 95%). This demographic process occurred around 7.8 ky ago (0.049–4.9x10^9^ HPD 95%). The convergence among MCMC chains and the ESS values behaved better for the N_e_ estimated, ancestral and present, than for T. This, along with the wide confidence intervals obtained for T, suggests that the latter parameter could not be properly estimated from our data, and both the median and intervals for T should be taken with caution.

## Discussion

The analyses of the polecat population in BPF before and after the arrival of the American mink, and of other additional populations with both microsatellite and mitochondrial markers suggested an almost absence of effects of the arrival of this non-native, invasive competitor on the genetic patterns of the polecat population in terms of both diversity and structure. This would be in agreement with recent data showing weak evidence that the American mink had a negative impact in the demography of the polecat in Poland [30].

### Diversity of the polecat population in Poland

Levels of nuclear diversity in the present polecat population in Poland are in the highest values of the range when compared to other polecat populations [25] and considerable higher than levels found in the impoverished British population [22]. This occurs in spite of the fact that half the markers used in our study were not specifically designed for the polecat, while those used in the British population were [22]. This situation was expected, given the critical status of the British population by the beginnings of the 20^th^ century [72]. Mitochondrial diversity in the polecat population from Poland is comparable to other polecat populations [25] although lower than in the British population, probably due to the hybridization with ferrets in the latter [22]. These levels of genetic diversity and the absence of any signal of a recent bottleneck in the Polish polecat population suggest that the population of polecats in Poland is not showing any sign of genetic decline, in contrasts with other populations in Europe (e.g. [25]).

### No effect of the arrival of the American mink in BPF on the polecat genetic diversity and an ancient population decline

We were not able to find differences in the levels of diversity between the extant population and the population predating the arrival of the American mink, at any of both nuclear or mitochondrial levels. Although the timeframe considered before and after the arrival of the American mink is not long, it still includes 53 generations, considering one year the generation time of the polecat [25]. Other studies including a smaller number of generations, using a similar number of markers and analysing temporal genetic variation have been able to detect changes in genetic diversity and/or structure patterns (e.g. [3, 35]). It is probable that the American mink affected the sex ratio of the polecat in BPF [30], as in other sites in Europe (e.g. [15, 26, 29]). Otherwise, our results would suggest that they did not erode the levels of genetic diversity of the polecat in BPF, at least in terms of neutral diversity. This is further supported by the fact that N_e_ has remained similar. Adult sex ratio (ASR) has proven to be heavily shifted towards males in NE Poland (Zalewski et al. in prep.) and in Belarus this shift being up to an increase from 59% to a 95% of males in the population [73]. Analysis of polecat sex ratio shift in Europe suggests that the invasion of American mink was a driver causing these changes [29]. The interaction with external factors causing an important decrease in the female population results in a demographic imbalance that may have negative consequences for the viability of the populations affected (e.g. [74]. Theory indicates that skewed sex ratio reduces effective population size (*N*_e_) and increases the deleterious effects of genetic drift. In the case of the polecat, one male mates with 1–3 females while females usually mate with one male [75]. If a significant reduction in the number of females occurred fewer males will be able to reproduce and thus contribute their genes to the following generation and this could affect levels of genetic diversity. This situation of reduced genetic diversity is not what we have found in the polecat population in BPF. It may be that there has not been enough time for the population to reach the equilibrium situation after the number of females were reduced and that any reduction in the levels of diversity would only be reflected in the future. However, since the arrival of the American mink to Eastern Poland in the late 70’s [34], enough polecat generations have passed (around 30 generations until 2012) to be able to detect changes in levels of diversity. Although our results suggested subtle allelic frequency changes around the 70’s, and that mitochondrial haplotype frequencies might have slightly changed, there is no clear pattern of differentiation between the population before and after the American mink invasion. Although very interesting and meriting further research, these observed changes in allelic frequencies at the end of the 60’s beginning of the 70’s could not be attributed to the arrival of the American mink. Mink arriving in the end of 70’s and its population increasing in the 80’s, the strongest competition between polecat and mink would be happening by the middle or the end of the 80’s [9]. It may also happen that the shift in the ASR in BPF was not strong enough to impact the genetic diversity of the population. On the other hand, even if the trophic niches of polecats and American mink are narrow and considerably overlap, they are able to reduce interspecies competition by using some mechanisms of food segregation [76]. This could act as an additional buffer on the impact of the invasion of the American mink in the polecat population.

On the other hand, the lack of genetic structure observed among populations from diverse areas of Poland, either as a result of a recent common origin of the potential populations or because they are connected by gene flow, would explain why genetic diversity could have been maintained. This lack of structure is remarkable given the geographical distance between the sampling locations (up to 700 km). The sex dispersing in the polecat is the male, while females remain more phylopatric [75]. They disperse mainly to mate in adjacent areas (the dispersal distance are not known), although in some cases they can move up to 35 km [77]. This gene flow may be favoured by the fact that polecats inhabit very different habitats, from riparian areas to forests and even villages [77]. This habitat plasticity would make the species more resilient to human occupation and the modification/fragmentation of the habitat, potentially allowing the connectivity among the different populations.

None of the methods used were able to detect a recent bottleneck in the polecat population. MSVAR was able to detect recent population reductions in otter and in the British polecat populations using a similar number of markers [22, 78], provided these reductions were important. This suggests that we can rule out the occurrence of a significant recent decrease in the polecat population, despite the wide HPD interval. The hunting bag of polecat in Poland increased from 300 in 1991–1992 to 2,400 polecats in 2007–2008, which may suggest an increase of polecat abundance, but this data should be treated with caution [79]. In accordance with our results and although accurate data on the census size of the polecat in Poland do not exist, indirect approaches suggest that the demographic changes suffered by the Polish and BPF polecat population have not been dramatic during the last century [30]. Otherwise, MSVAR detected an ancient bottleneck, a reduction to one sixth of the Polish polecat population occurring several thousand years ago. This coincides in time with the arrival of the Neolithic in Central Europe, which implied an important increase in human population [80]. The planet was already largely transformed by 3,000 years ago by humans [81], and some European mammals suffered range losses and significant declines starting around this time, including mustelids [82] and polecats may have been one of these species affected. Although polecats may adapt to very different habitat, including anthropized ones, they are still affected by human activities at present in some regions [83]. However, this demographic reduction was not detected with the analysis of the mitochondrial fragment. Mitochondrial DNA is expected to be more sensitive to changes in demography due to its ¼ N_e_ with respect to the nuclear genome. Otherwise, confidence intervals are big enough to infer that the bottleneck happened earlier in time, and the mtDNA fragment analysed is not informative enough to trace it. A bigger battery of nuclear markers as SNPs would be necessary to have a clearer picture on this question.

### Conservation implications

A review on the polecat status in 34 countries found that populations were declining or suspected to be declining in 20 of them, including Poland [84]. However, in several cases the information based on expert opinion but not supported by the data, was insufficient to assess the status of the population [85], including status of polecats in Poland. Our results are positive news for the species in this area of Europe. Genetic diversity of the species in Poland seems not to be altered after the arrival of the American mink, probably due to this high level of connectivity among the populations as discussed above, and the partial habitat segregation of both species. This suggests that the Polish population has been genetically stable in the recent history. Additionally, the absence of ferrets in the area [33] make the hybridization with these close relatives impossible, as seen in other populations [22, 72], thus the integrity of the species is not threatened. Both aspects make us suggest the polecat population in Białowieża Primeval Forest and eventually, the Polish population, as a source for captive breeding and/or reintroductions in other areas where the species has been depleted as in the UK, provided the adequate genetic and ecological studies are conducted, including an assessment of potential local adaptations. Otherwise, a thorough study on the intensity of the population skew on the ASR and its potential genetic effects should be conducted before any management action is taken in this sense. Additionally, although our data suggest that the invasion of the American mink has had little genetic effect in the polecat population in Eastern Poland, it would be interesting to develop a significantly larger battery of markers, as thousands of SNPs along the genome, to fully understand the genetic effects of the invasion of the American mink in the patterns of genetic diversity of the European polecat population in Poland.

## Supporting information

S1 Fig Evolution of the effective population size N_e_ of the polecat population in Poland using the software BEAST. (PDF)

S1 Table Complete list of samples used in this study. (XLSX)

S2 Table Sliding-window cohorts for A- microsatellites and B- mtDNA sequences. Samples were subdivided in cohorts of four years. n indicates the number of individuals in each cohort. Only cohorts integrated by eight or more individuals are listed and considered for the analyses (and thus included here).
(XLSX)

S3 Table Description of the microsatellites tested and used, and tested but not used in this study. A) Number of alleles, He and Ho values for the eleven microsatellites for all the samples in this study. B) List of microsatellite markers tested but not finally used in this study.
(XLSX)

S4 Table Microsatellite genotypes of the individuals used in this study. (XLSX)

S5 Table A- mtDNA haplotypes found in this study. B- Distribution of the Haplotypes in the populations.
(XLSX)

S6 Table FST values among populations estimated with the 11 microsatellite markers. (XLSX)

S7 Table Phist, and Phist and pairwise differences p-values among populations estimated with the fragment of mitochondrial control region sequenced in this study. (XLSX)
