# Supplementary figures and images for: The genetic diversity and structure in the European polecat were not affected by the introduction of the American mink in Poland
Source: PLoS One. 2022 Sep 28;17(9):e0266161. doi: 10.1371/journal.pone.0266161 (PMC9518895; doi:10.1371/journal.pone.0266161)

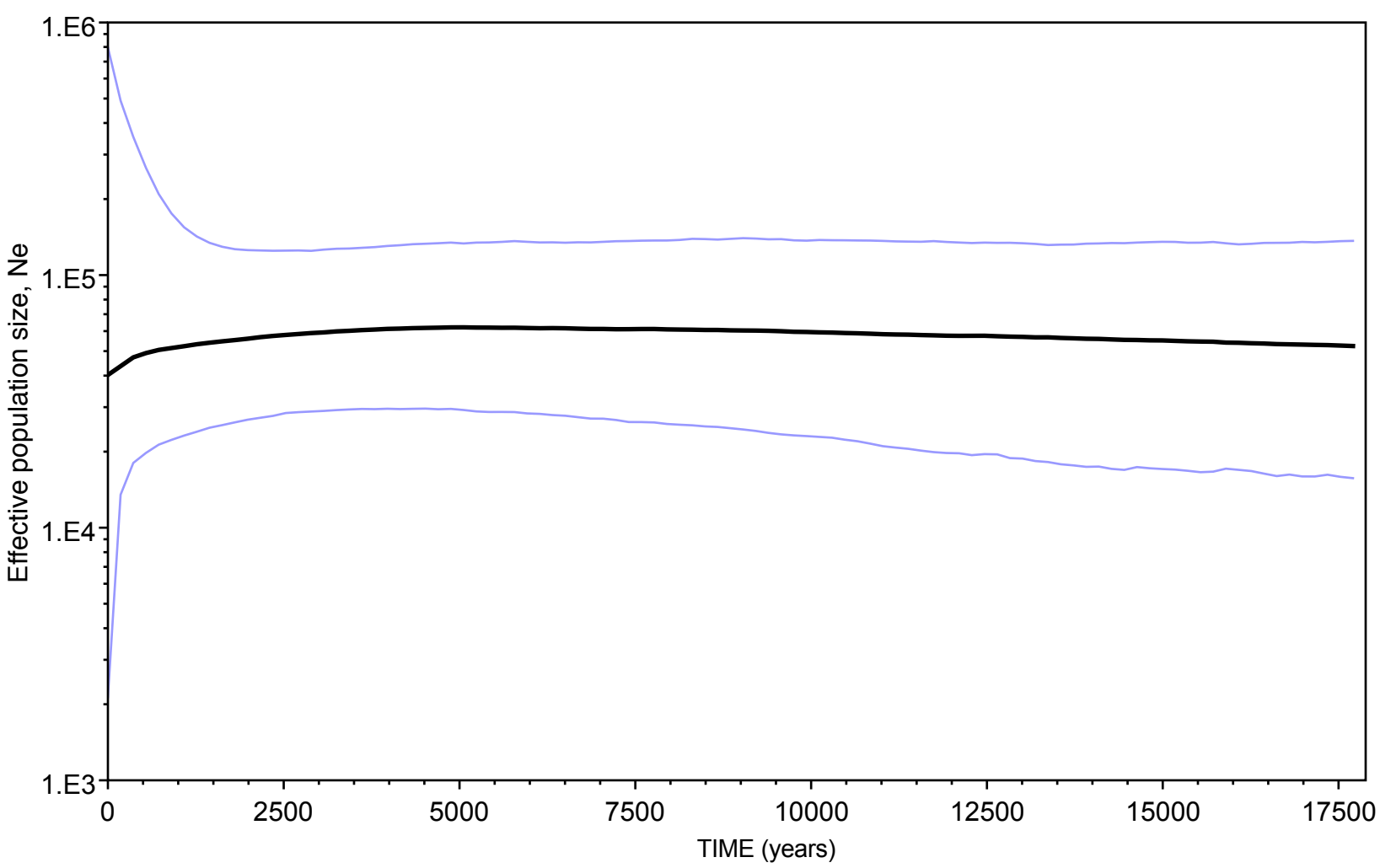

Supplement: S1 Fig — (PDF) [file pone.0266161.s001.pdf]
